# Forty-five per cent lower acute injury incidence but no effect on overuse injury prevalence in youth floorball players (aged 12–17 years) who used an injury prevention exercise programme: two-armed parallel-group cluster randomised controlled trial

**DOI:** 10.1136/bjsports-2019-101295

**Published:** 2020-01-28

**Authors:** Ida Åkerlund, Markus Waldén, Sofi Sonesson, Martin Hägglund

**Affiliations:** 1 Department of Health, Medicine and Caring Sciences, Unit of Physiotherapy, Linköping University, Linköping, Sweden; 2 Department of Health, Medicine and Caring Sciences, Sport Without Injury ProgrammE (SWIPE), Linköping University, Linköping, Sweden; 3 Department of Health, Medicine and Caring Sciences, Unit of Public Health, Linköping University, Linköping, Sweden; 4 Department of Orthopaedics, Hässleholm-Kristianstad-Ystad Hospitals, Hässleholm, Sweden

**Keywords:** adolescent, exercises, injury prevention, intervention efficacy, randomised controlled trial

## Abstract

**Objective:**

To study whether an injury prevention exercise programme would reduce the number of injuries in youth floorball players.

**Methods:**

81 youth community level floorball teams (48 clusters=clubs) with female and male players (12–17 years) were cluster-randomised into an intervention or control group. Intervention group coaches were instructed to use the Swedish *Knee Control* programme and a standard running warm-up before every training session, and the running warm-up before every match, during the season. Control teams continued usual training. Teams were followed during the 2017/2018 competitive season (26 weeks). Player exposure to floorball and occurrence of acute and overuse injuries were reported weekly via a web-based player survey using the Oslo Sports Trauma Research Centre Questionnaire.

**Results:**

17 clusters (301 players) in the intervention group and 12 clusters (170 players) in the control group were included for analyses. There were 349 unique injuries in 222 players. The intervention group had a 35% lower incidence of injuries overall than the control group (adjusted incidence rate ratio (IRR) 0.65, 95% CI 0.52 to 0.81). The absolute risk reduction was 6.6% (95% CI 3.2 to 10.0), and the number needed to treat was 152 hours of floorball exposure (95% CI 100 to 316). Intervention group teams had a 45% lower incidence of acute injuries (adjusted IRR 0.55, 95% CI 0.37 to 0.83). There was no difference in the prevalence of overuse injuries (adjusted prevalence rate ratio 0.96, 95% CI 0.73 to 1.26).

**Conclusion:**

The *Knee Control* injury prevention programme reduced acute injuries in youth floorball players; there was no effect on overuse injuries.

**Trial registration number:**

Clinical Trials NCT03309904.

## Introduction

Floorball is a pivoting sport played with a plastic stick and light plastic ball on a 40×20 m sized pitch with five field players and one goalkeeper in each team. Free player interchanges are allowed. The sport demands quick running with sudden stops, accelerations/decelerations, and sharp changes of direction on an indoor playing surface made of rubber or parquet. Sixty-eight countries are affiliated with the International Floorball Federation.^1^ There are few studies investigating injury risk in floorball, particularly among youth players.^2^ Injury incidence in junior and adult floorball is as high as in other popular team ball sports.^3–5^

To date, only two studies have evaluated injury prevention interventions in floorball, both in elite adult players.^6 7^ A 66% reduction in acute non-contact lower limb injuries was seen in females in Finland using a neuromuscular training programme.^6^ No injury preventive effect was seen after introducing a cognitive behavioural theory based psychological intervention in males and females in Sweden.^7^

The Swedish injury prevention exercise programme *Knee Control* reduced the rate of anterior cruciate ligament (ACL) injuries by 64% in female youth football players.^8^ Other programmes with similar exercise content reduced the risk of overall acute and overuse injuries in other pivoting team sports such as football,^9 10^ basketball^11^ and handball.^12^ The primary aim of this cluster randomised controlled trial (RCT) was to study the *Knee Control* injury prevention programme and a standard running warm-up to reduce the number of injuries in youth floorball players compared with usual training.

## Methods

### Study design

This two-armed parallel-group cluster RCT was registered with Clinical Trials (NCT03309904) (submitted 10 October, posted 16 October 2017). Study protocols and reporting adhered to the Consolidating Standards for Reporting Clinical Trials (CONSORT) statement.^13^ The intervention was implemented in a team setting, therefore a cluster design was chosen to minimise contamination between the intervention and control groups. A cluster was defined as all male or female teams registered in the same club.

### Recruitment

We recruited youth teams participating at community level in two regional districts of the Swedish Floorball Federation (Småland and Östergötland). In August 2017, before the start of the season, the Swedish Floorball Federation sent an invitation email to all eligible teams in the two districts. The research group then approached the coaches of all teams over telephone to provide additional information about the study and the inclusion criteria for final recruitment. Three-hundred and forty teams were registered for the 2017–2018 season and were assessed for eligibility. The inclusion criteria were: (1) team included players aged 12–17 years; (2) team had not used any injury prevention exercise programme regularly in the last year; and (3) team had scheduled ≥2 team training sessions per week during the season. The exclusion criteria were: (1) team with most of their players outside the age range; and (2) individual players in included teams who were outside the age range.

### Randomisation

**Figure 1:**
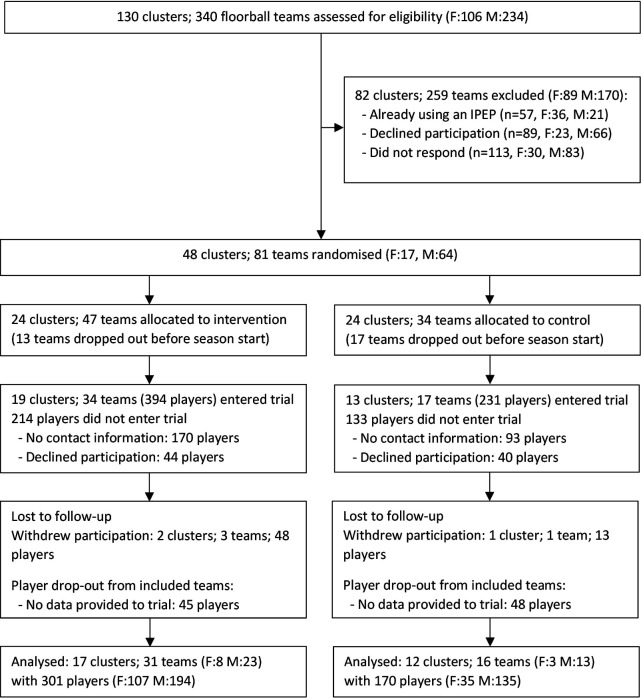
Flowchart of clusters (clubs), teams and players throughout the trial. F, female; IPEP, injury prevention exercise programme; M, male.

All teams that met the inclusion criteria and agreed to participate were randomised into an intervention or control group by cluster (figure 1). A statistician performed the randomisation with a computer-generated list of random numbers, stratified by district and sex. The statistician was not blinded at this stage to ensure all teams within the same club were included in the same cluster and randomised to the same group. Randomisation was revealed to teams after recruitment to ensure concealment of allocation. Due to the nature of the training intervention, coaches and players were not blinded to group allocation. Final player inclusion was based on informed consent from each player (and legal guardians if the player was <15 years of age). All players and their legal guardians received written and oral information about the study.

### Intervention

The *Knee Control* injury prevention programme (Knäkontroll, SISU Idrottsböcker, Sweden, 2005) focuses on lower limb and core strength, neuromuscular control, balance, and jumping and landing technique. The programme has previously been evaluated in youth football,^8^ and consists of six principal exercises; one legged knee squat, pelvic lift, two legged knee squat, the bench, the lunge, and jumping/landing technique (see online supplementary table 1). Each exercise has three to four steps of progression with increasing difficulty and one partner exercise for variation. All teams were recommended to start at exercise level A (easiest) and then progress (usually the whole team) to the next level when the coach assessed that the players’ strength, balance and neuromuscular control improved.

10.1136/bjsports-2019-101295.supp1 Supplementary data

The intervention consisted of a standardised 5 min running warm-up (see online supplementary table 1), followed by the *Knee Control* intervention with three sets of 8–15 repetitions for each exercise (10–15 min). The teams in the intervention group were instructed to use the running warm-up and the *Knee Control* programme before every training session throughout the season and to use the running warm-up before all matches. The control group teams were instructed to train and play as usual.

### Pre-study workshops and follow-up

The study methods and reporting procedures were presented to all coaches at information workshops before the start of the season. For the intervention group, a study physiotherapist educated coaches on the intervention rationale and held a practical workshop of the exercises together with coaches and two invited players from each team. Coaches were then responsible for carrying out the intervention with their teams during the season. To help implement *Knee Control*, written and oral instructions were given on how to deliver the programme and instruct the players, and leaflets and videos of all exercises were provided. A study physiotherapist visited teams in the intervention group who could not attend the pre-season workshops and gave the same study information and intervention instructions. A member of the study group (physiotherapist or final-year physiotherapy student trained in *Knee Control*) visited teams in the intervention group once during the first half and once during the second half of the season to assist with any difficulties in performing *Knee Control* and to increase intervention compliance and fidelity (28 of 31 teams visited; three teams cancelled the training session with short notice when the visit was planned). The physiotherapist gave feedback on how to perform the exercises and answered any questions from the coaches. Control group coaches who could not attend the pre-season workshops were given oral and written study information by telephone and email.

### Baseline data collection and monitoring of injuries

Data were collected during one competitive season of 26 weeks (1 October 2017 to 31 March 2018). Data from teams in the intervention group were included in the study from the date of their first *Knee Control* instruction if this was later than 1 October (seven teams had up to 2 weeks delayed start; median 1 week). Players answered a baseline questionnaire including for instance demographics, previous floorball experience, participation in other sports, and previous use of *Knee Control*.^14^

Players also answered a weekly survey administered via a web-link through a short messaging service (SMS) and email to their mobile phone sent at 18:00 hours each Sunday. If the players did not complete the survey on the same or the following day, two additional reminders were sent via SMS on the Tuesday and Thursday. Online software (esMakerNX3 V3) was used for administration of the survey. The players had to answer a minimum of four and maximum of 22 questions, taking 1–10 min to complete (see online supplementary figure 1). Survey questions included match and training exposure and occurrence of any injury in the past week. If the player reported full participation in floorball without any health problems the questionnaire ended. If an injury was reported the player answered the four questions of the Oslo Sports Trauma Research Centre (OSTRC) Overuse Injury Questionnaire to evaluate its impact on sports participation, training volume, performance and pain.^15^ Questions on time loss from floorball, medical attention, location and type of injury were also included. For all time loss and medical attention injuries a physiotherapist from the research group contacted the player via telephone for further information about the injury using a standardised injury report form. Injury definitions used in the study are presented in box 1.

10.1136/bjsports-2019-101295.supp2 Supplementary data

Box 1 **Injury definitions**

**Injury:** Any physical complaint sustained by a player that results from floorball training or a match, irrespective of the need for medical attention or time loss from floorball activities.^30^

**Acute injury:** Injury that occurs suddenly and is associated with a specific, identifiable event.^30^

**Overuse injury:** Injury caused by repeated microtrauma without a single, identifiable event responsible for the injury, often with gradual onset.^30^

**Substantial injury:** Injury leading to moderate or severe reductions in training volume or performance, or inability to participate in floorball.^15^

**Time loss injury:** Injury that causes absence from floorball training or match play.^30^

**Medical attention injury:** Injury that results in the player receiving medical attention^30^ (eg, primary care, emergency room or hospital care).

**Reinjury:** Injury of the same type and at the same site as an index injury that the player has suffered previously in the current or in the preceding season.^30^

**Injury event:** Any new injury or recurrent injury after the player has reported at least 1 week of full floorball participation without any health problems between the index injury and the recurrence. Multiple consecutive weeks of the same reported health problem (eg, several weeks of time loss from play or several weeks affected training volume, performance, participation or pain) were considered as the same injury event.

In addition to player reporting, coaches reported playing time (minutes of participation in training and match) and absence (due to injury, illness or other reasons) for each player on a computer-based exposure form,^8^ and emailed data to the research group each month. Coaches in the intervention group also documented compliance with the 5 min running warm-up and *Knee Control* programme for training sessions and matches on the exposure form. If any information was missing or unclear, coaches were contacted to correct the data throughout the season.

### Outcome measures

The primary outcome was the incidence and prevalence of all reported floorball injuries. Secondary outcomes were time loss injuries and medical attention injuries. We made a deviation from the registration protocol and also report rates of acute injuries, overuse injuries, substantial injuries, lower limb and knee injuries separately as secondary outcomes. Injuries were captured via the weekly player report and additional information regarding time loss from floorball was obtained from the standardised injury report form and the coach exposure form.

Injury incidence is expressed as the number of unique injury events (including new and recurrent injuries) per 1000 hours of floorball play. Injury incidence is presented for all injuries as the primary outcome and reported separately for lower limb, knee, time loss and acute injuries.

Weekly injury prevalence is expressed as the number of player reports where a player reported a floorball injury divided by the total number of eligible player reports each week. An eligible weekly player report was defined as a week with floorball exposure or where the player reported total absence because of a floorball injury. Injury prevalence is presented for all injuries as the primary outcome, and for lower limb, knee, substantial and overuse injuries specifically.

### Sample size

We calculated the sample size for the primary outcome of any reported injury and estimated that 50%^16^ of players would report at least one injury during the season and a 40% reduction in injury rate in the intervention group.^10^ With adjustment for a cluster effect (inflation factor 1.28) the required sample was 403 participants to provide a minimum of 80% power (α=0.05). We accounted for an estimated drop-out rate of 30% and therefore aimed to recruit at least 524 players (approximately 40 teams).

### Statistical methods

Generalised estimating equations were used to analyse the cluster-aggregated weekly data. We applied a Poisson distribution with a log link function, and model-based SE estimation, and by assuming a first-order autoregressive working correlation structure within cluster, to calculate the rate ratio and corresponding 95% CI for the injury incidence and prevalence according to the intention to treat (ITT) principle. The ITT analyses were adjusted for sex, and all analyses were also reported by sex separately. The statistician who performed the ITT analysis was blinded to group allocation. We calculated the number needed to treat (NNT) as the inverted absolute rate reduction (ARR) for the primary outcome. All analyses were performed using SPSS statistical software for Windows (v25; IBM, New York). We considered a p value <0.05 to be significant.

Injury incidence is graphically presented as a moving average, using the average injury incidence over two consecutive weeks (eg, moving average week 2=competitive week 1+2) to smooth out spikes in weekly incidences. Team intervention compliance is expressed as the proportion of the total number of training sessions and matches where the coach reported using *Knee Control* and the running warm-up over the season. Player intervention compliance is expressed as the weekly mean (SD) number of *Knee Control* sessions over the season. No imputation was made for missing data (eg, missing weekly player reports).

### Patient and public involvement

The Swedish Floorball Federation and the Floorball Competence Centre (Innebandyns Kompetenscentrum) at Umeå University were included in the planning and preparation of this study. Their involvement included input on the study plan, surveys and research questions, help with recruitment of participants, and plans for dissemination of the study results to participants and wider relevant communities. They had no influence on the analyses, interpretation of results or manuscript preparation.

## Results

Forty-eight clusters with 81 teams and 1500 players were randomised. Thirteen teams in the intervention group and 17 teams in the control group dropped out shortly after randomisation before the season started, and an additional four teams and another 440 players (from included teams) were lost to follow-up. In total, 29 clusters with 47 teams and 471 players were included in the analyses (figure 1). Mean (SD) age was 13.6 (1.1) years (boys 13.5, girls 13.8) in the intervention group, and 13.2 (1.3) years (boys 13.1, girls 13.6) in the control group (see online supplementary table 2).

10.1136/bjsports-2019-101295.supp3 Supplementary data

A total of 7503 weekly player reports were registered, with an average weekly response rate of 64% (95% CI 63% to 65%) in the intervention group and 58% (95% CI 57% to 60%) in the control group.

### Exposure, compliance and floorball injury characteristics

Total weekly player floorball exposure in the intervention group was 3.95 hours (1.09 match; 2.89 training) and in the control group 3.81 hours (1.10 match; 2.80 training) (p=0.074). Team compliance with the running warm-up and *Knee Control* in the intervention group was 84% (range 13–100%) of training sessions (females 83% (13–100%), males 85% (47–100%)). Team compliance with the running warm-up before matches was 53% (range 0–100%). Players in the intervention group performed a mean (SD) of 1.45 (1.02) *Knee Control* sessions per week during the season (females 1.34 (1.06), males 1.51 (0.99]; p<0.001). There were 349 unique injuries reported in 222 (47%) players (table 1).

**Table 1:** Characteristics of all floorball injuries

	Intervention group			Control group		
	Total (n=197)	Acute (n=52)	Overuse (n=145)	Total (n=152)	Acute (n=47)	Overuse (n=105)
Time loss, n (%)	79 (40)	28 (54)	51 (35)	64 (42)	25 (53)	39 (37)
Medical attention, n (%)	43 (22)	14 (27)	29 (20)	23 (15)	6 (13)	17 (16)
New injury, n (%)	87 (44)	40 (77)	47 (32)	62 (41)	27 (57)	35 (33)
Reinjury, n (%)	110 (56)	12 (23)	98 (68)	90 (59)	20 (43)	70 (67)
Injury type, n (%)						
Fractures		1 (2)			0 (0)	
Joint and ligament		15 (29)			17 (36)	
Muscle and tendon		15 (29)			12 (26)	
Contusions		16 (31)			15 (32)	
Lacerations and skin		0 (0)			0 (0)	
CNS and PNS		0 (0)			0 (0)	
Other	150 (77)	5 (10)	145 (100)	108 (71)	3 (6)	105 (100)
Injury location, n (%)*						
Lower limbs	155 (78)	40 (77)	115 (79)	126 (83)	40 (85)	86 (82)
Hip/groin	13 (7)	6 (12)	7 (5)	7 (4)	5 (11)	2 (2)
Thigh	15 (8)	11 (21)	4 (3)	12 (8)	7 (15)	5 (5)
Knee	87 (44)	9 (17)	78 (54)	57 (38)	7 (15)	50 (48)
Lower leg/Achilles tendon	13 (7)	3 (6)	10 (7)	16 (11)	7 (15)	9 (9)
Ankle	10 (5)	8 (15)	2 (1)	18 (12)	12 (26)	6 (6)
Foot/toe	17 (9)	3 (6)	14 (10)	16 (10)	2 (4)	14 (13)
Trunk	16 (8)	5 (10)	11 (8)	9 (6)	4 (9)	5 (5)
Upper limbs	12 (6)	4 (8)	8 (6)	10 (7)	3 (6)	7 (7)
Head and neck	6 (3)	3 (6)	3 (2)	1 (1)	0 (0)	1 (1)
Other	1 (0.5)	0 (0)	1 (1)	2 (1)	0 (0)	2 (2)

*Injury location missing for seven injuries in the intervention group and four in the control group.

CNS, central nervous system; PNS, peripheral nervous system.

### Intervention effect on the incidence of floorball injuries

**Figure 2:**
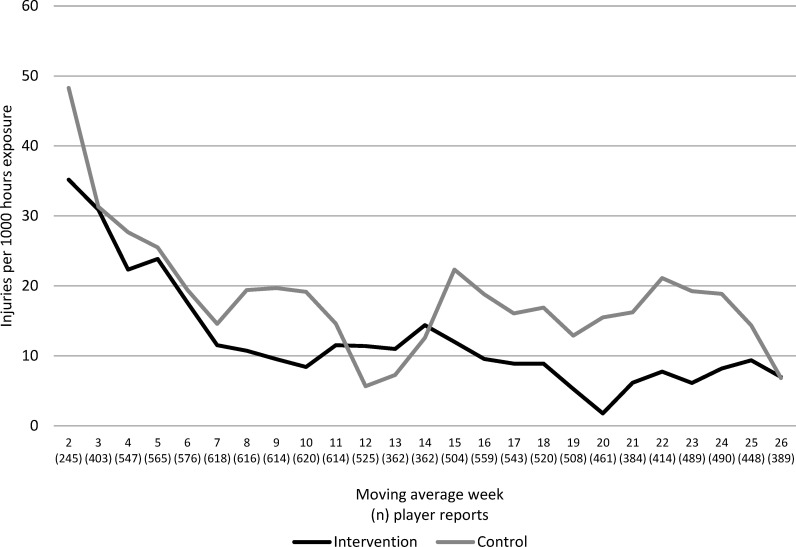
Sex-adjusted injury incidence per week, based on moving average (moving average week 2=competitive week 1+2; moving average week 3=competitive week 2+3, etc) in the intervention group (black line) and control group (grey line). Values within parentheses on the x axis show the number of weekly player reports for each moving average week observation.

Weekly injury incidences for both groups are shown in figure 2. The intervention group had a 35% lower incidence of injuries overall (adjusted incidence rate ratio (IRR) 0.65, 95% CI 0.52 to 0.81) and a 45% lower incidence of acute injuries (adjusted IRR 0.55, 95% CI 0.37 to 0.83) compared with the control group (table 2). The ARR for all injuries was 6.6% (95% CI 3.2 to 10.0), and the NNT was 152 hours of floorball exposure (95% CI 100 to 316), meaning that to prevent one injury approximately three players must perform the *Knee Control* programme during one season.

**Table 2:** Number and incidence (injuries per 1000 playing hours) of all and acute floorball injuries and incidence rate ratio in the intervention group versus control group

	Intervention group N, incidence (95% CI)	Control group N, incidence (95% CI)	Incidence rate ratio (95% CI)	P value
All injuries				
Total*	197, 12.1 (10.4 to 14.0)	152, 18.7 (15.8 to 22.1)	0.65 (0.52 to 0.81)	**<0.001**
Female	76, 12.8 (10.0 to 16.3)	31, 16.6 (11.2 to 24.4)	0.77 (0.49 to 1.22)	0.265
Male	121, 11.7 (9.8 to 14.1)	121, 19.3 (16.1 to 23.2)	0.61 (0.47 to 0.79)	**<0.001**
Lower limb injuries				
Total*	155, 9.5 (8.1 to 11.2)	126, 15.5 (12.9 to 18.6)	0.62 (0.48 to 0.79)	**<0.001**
Female	58, 9.7 (7.3 to 12.9)	27, 14.4 (9.5 to 21.7)	0.68 (0.41 to 1.12)	0.126
Male	97, 9.4 (7.7 to 11.5)	99, 15.8 (12.9 to 19.3)	0.60 (0.45 to 0.79)	**<0.001**
Knee injuries				
Total*	87, 5.4 (4.3 to 6.7)	57, 7.0 (5.4 to 9.2)	0.76 (0.54 to 1.08)	0.127
Female	34, 5.8 (4.0 to 8.3)	12, 6.3 (3.4 to 11.7)	0.91 (0.45 to 1.86)	0.798
Male	53, 5.2 (3.9 to 6.8)	45, 7.2 (5.3 to 9.7)	0.72 (0.48 to 1.08)	0.108
All time loss injuries				
Total*	79, 4.9 (3.9 to 6.1)	64, 7.7 (6.0 to 9.9)	0.64 (0.45 to 0.90)	**0.010**
Female	28, 4.6 (3.3 to 6.5)	9, 4.8 (2.6 to 8.8)	0.96 (0.48 to 1.94)	0.916
Male	51, 4.9 (3.7 to 6.6)	55, 8.7 (6.6 to 11.5)	0.57 (0.38 to 0.85)	**0.005**
Acute injuries				
Total*	52, 3.2 (2.4 to 4.2)	47, 5.8 (4.3 to 7.7)	0.55 (0.37 to 0.83)	**0.004**
Female	21, 3.5 (2.3 to 5.4)	9, 4.8 (2.5 to 9.3)	0.73 (0.33 to 1.60)	0.431
Male	31, 3.0 (2.1 to 4.3)	38, 6.0 (4.4 to 8.4)	0.50 (0.31 to 0.81)	**0.005**
Acute time loss injuries				
Total*	28, 1.7 (1.2 to 2.5)	25, 3.1 (2.1 to 4.5)	0.55 (0.33 to 0.94)	**0.029**
Female	11, 1.8 (1.0 to 3.2)	6, 3.2 (1.5 to 6.9)	0.57 (0.22 to 1.50)	0.254
Male	17, 1.7 (1.0 to 2.6)	19, 3.0 (2.0 to 4.7)	0.54 (0.29 to 1.03)	0.062

P values in bold type are statistically significant.

*Model adjusted for sex.

### Intervention effect on prevalence of floorball injuries

**Figure 3:**
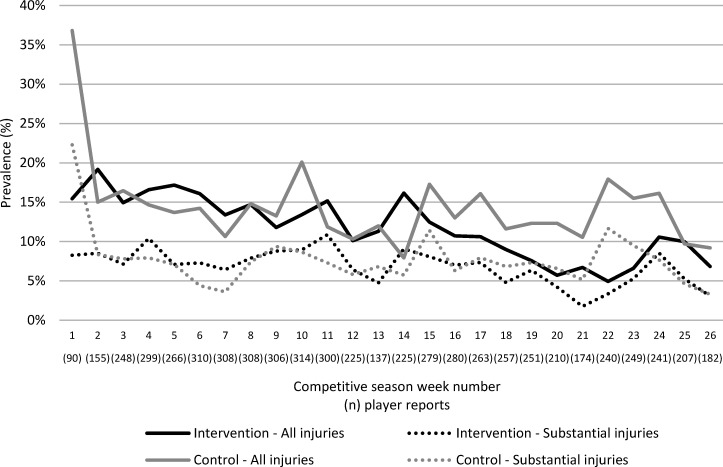
Sex-adjusted weekly injury prevalence in the intervention group: all injuries (black line) and substantial injuries (black dotted line); control group: all injuries (grey line) and substantial injuries (grey dotted line). Values within parentheses on the x axis show the number of weekly player reports for each observation.

Weekly injury prevalence is illustrated in figure 3. There was a 15%, non-statistically significant, lower weekly prevalence of any floorball injury in the intervention group compared with the control group (adjusted prevalence rate ratio 0.85, 95% CI 0.68 to 1.07) with an average weekly prevalence difference of 2.1% (95% CI 0.2 to 4.0). No difference in weekly prevalence of overuse injuries was seen. Females in the intervention group had a statistically significant higher prevalence of knee injuries compared with the control group (table 3). A majority of knee injuries in females were overuse injuries (28 of 34 knee injuries in the intervention group, 10 of 12 in the control group) with the remaining being muscle/tendon (intervention 3, control 1) joint/ligament (intervention 2, control 1) and contusion (intervention 1).

**Table 3:** Weekly prevalence of floorball injuries, and prevalence rate ratio in the intervention versus control group

	Prevalence intervention group (95% CI)	Prevalence control group (95% CI)	Prevalence rate ratio (95% CI)	P value
All injuries				
Total*	12.0 (10.5 to 13.8)	14.1 (11.8 to 16.8)	0.85 (0.68 to 1.07)	0.168
Female	14.5 (11.9 to 17.8)	16.5 (11.8 to 23.1)	0.88 (0.60 to 1.30)	0.520
Male	11.0 (9.2 to 13.3)	13.1 (10.7 to 16.2)	0.84 (0.64 to 1.11)	0.218
Lower limb injuries				
Total*	9.6 (8.2 to 11.3)	11.6 (9.4 to 14.2)	0.83 (0.64 to 1.08)	0.160
Female	12.1 (10.5 to 14.0)	12.5 (9.7 to 15.9)	0.98 (0.74 to 1.29)	0.861
Male	8.5 (6.9 to 10.5)	11.1 (8.8 to 14.0)	0.77 (0.56 to 1.05)	0.100
Knee injuries				
Total*	6.4 (5.2 to 7.9)	5.2 (3.8 to 7.1)	1.24 (0.84 to 1.82)	0.275
Female	8.4 (6.4 to 11.0)	3.3 (1.5 to 7.2)	2.52 (1.11 to 5.72)	**0.027**
Male	5.4 (4.1 to 7.2)	5.7 (4.0 to 8.1)	0.95 (0.60 to 1.49)	0.823
Substantial injuries				
Total*	7.1 (6.0 to 8.5)	7.5 (5.9 to 9.5)	0.96 (0.71 to 1.29)	0.761
Female	8.7 (7.0 to 10.9)	7.6 (5.0 to 11.5)	1.15 (0.71 to 1.84)	0.573
Male	6.5 (5.1 to 8.2)	7.3 (5.5 to 9.7)	0.89 (0.61 to 1.30)	0.546
Overuse injuries				
Total*	9.5 (8.0 to 11.1)	9.9 (7.9 to 12.3)	0.96 (0.73 to 1.26)	0.762
Female	11.2 (8.8 to 14.2)	8.6 (5.3 to 13.9)	1.31 (0.76 to 2.25)	0.328
Male	8.6 (6.9 to 10.6)	10.2 (8.0 to 13.0)	0.84 (0.61 to 1.16)	0.296
Substantial overuse injuries				
Total*	5.1 (4.1 to 6.3)	5.6 (4.2 to 7.5)	0.91 (0.63 to 1.31)	0.606
Female	6.1 (4.6 to 8.1)	4.9 (2.8 to 8.6)	1.25 (0.66 to 2.37)	0.493
Male	4.6 (3.4 to 6.2)	5.8 (4.1 to 8.0)	0.80 (0.51 to 1.25)	0.324

Substantial injuries were those that lead to moderate or severe reductions in training volume or performance, or inability to participate in floorball.

P values in bold type are statistically significant.

*Model adjusted for sex.

## Discussion

Our main finding was that youth floorball teams that used the *Knee Control* injury prevention programme had a 35% lower overall incidence of injuries and 45% lower incidence of acute injuries compared with control group teams that performed their usual training.

### Intervention effect on injury incidence and acute injuries

Our findings expand on previous studies showing a similarly lower rate of lower limb injuries in pivoting team sports using an injury prevention programme.^6 8 10–12^ There were lower injury rates in the current study for acute injuries and for males. One possible explanation is that male players received a higher dosage of *Knee Control* during the season, and higher programme compliance correlates with larger preventive effects.^17–19^

For specific injury types such as ACL injuries, lower rates of injury (more effective intervention) are typically reported for females.^20^ Our study was not designed for ACL injuries specifically, and is under-powered for such analyses. Incidence rate ratios for females were 0.77 for all injuries and 0.68 for lower limb injuries; this was not statistically significant—likely due to the subgroup analyses being under-powered. A 20–30% lower rate of injuries in the intervention group is of clinical interest and shows that the *Knee Control* programme should be used in female as well as in male youth floorball players. To complement the mainly low-tempo and controlled exercises of the *Knee Control* programme, for the intervention to serve as a complete pre-training and match warm-up, we added a 5 min running warm-up. This standardised running warm-up was not part of our previous *Knee Control* RCT in football,^8^ but has since been included in the commercially available version of *Knee Control*.

### Intervention effect on injury prevalence and overuse injuries

There was a smaller and non-statistically significant preventive effect on injury prevalence for injuries overall and no preventive effect on injury prevalence for overuse injuries. For females we even found a possible adverse intervention effect on knee injuries (two times higher prevalence rate in the intervention group), with >80% being overuse injuries. Specific diagnoses are not available from athlete self-reports, but patellofemoral pain is a common overuse syndrome in youth female athletes.^21 22^ Activities that load the patellofemoral joint such as squatting and running are potential aggravating factors,^23^ and some exercises in *Knee Control* (lunges, one and two legged knee squats) might provoke symptoms in some athletes with patellofemoral pain.

Teaching coaches how to instruct on correct exercise execution, how to provide feedback to players, and how to modify exercises if necessary, could help reduce such adverse outcomes. This is important to ensure high adherence to the prevention programme *Knee Control*. Further study is also warranted on measures to reduce the burden of overuse injury in young athlete populations. Two-thirds of self-reported injuries in our floorball population were overuse-related, with similar findings reported from football where 47% of youth players aged 9–14 years experienced an overuse injury during a season, with a weekly prevalence of 12.8%.^24^

### Seasonal variations in injury rates

Injury incidence and prevalence were highest at the beginning of the season in both groups, which extends previous findings in handball.^25^ Many players in our youth cohort participate in other sports, mainly football, and began the season with an injury.^14^ Early season injuries can also be explained by a sudden change in training load and sport exposure (eg, running characteristics and change of surface). Any faulty kinematics present at the start of the season could also result in greater injury rates at this period. As evident from figures 2 and 3 the injury incidence and prevalence dropped during the season in the intervention group, possibly as a result of neuromuscular adaptation from the injury prevention programme.

As some of the teams started the competitive season later than 1 October, the study sample was smaller in the first weeks of the season, resulting in less robust estimates. Possibly, a drop in injury rates due to declining response proportions should also be considered, as reported in a previous study.^25^

### Strengths and limitations

Strengths of this trial are the high (>80%) compliance with the *Knee Control* injury prevention programme, the cluster design to minimise contamination between the intervention and control groups, and player self-reporting of injuries using a validated questionnaire,^15^ which also captures non-time loss injuries. Validity of self-reported injuries in this youth cohort is not known, but we collected additional injury information via telephone for time loss and medical attention injuries. The same method has been used in similar studies in youth athletes.^25–27^

We deviated from the original OSTRC questionnaire protocol in that for players who reported “full participation without health problems” on the first question of the questionnaire the survey ended, and questions 2–4 of the questionnaire were set to severity score 0 (no reduction training volume, no reduced performance, no pain related to floorball). This was done to minimise the survey response burden each week. We believe our procedures are still valid since we should not expect players to report any health problems on questions 2–4 of the questionnaire if they stated full participation without health problems on question 1. This procedure is also in accordance with the 2020 update of the OSTRC questionnaire application guidelines.^28^

The low response rate was a limitation, but is comparable with previous studies in youth athletes (60–66%).^27 29^ A previous study on elite youth athletes reported a higher response rate (92%).^25^ Measures to improve the response rate in the current trial included autogenerated SMS reminders and encouragement of coaches to remind players to answer the weekly survey.

Another limitation was the high drop-out rate after randomisation, possibly because of a perceived burden to participate in an RCT. Higher drop-out rate in the control group may reflect dissatisfaction with the randomisation outcome, despite control group teams being offered injury prevention education and materials after the season.

Sample size was sufficient for the main outcome, but we were under-powered for some analyses of secondary outcomes, and also for some sex-specific analyses, especially in females. We have no obvious reason to believe that teams and players who dropped out were systematically different from the included teams, and thus believe the positive injury preventive effects of *Knee Control* are generalisable to the Swedish population of youth floorball players. Our sample comprised a mix of sexes and ages (range 12–17 years) with young athletes at varying stages of both physical and mental development. The age distribution was similar in the intervention and control group and should thus not influence the intervention effect comparisons. As the proportion of females in the intervention group was greater than in the control group (36% vs 21%) all analyses of the total population were sex-adjusted, and outcomes presented for males and females separately. As we made a large number of statistical group comparisons the risk of false positives should be considered.

## Conclusion

Youth floorball teams that used the *Knee Control* injury prevention programme had a 35% reduction in injury incidence overall and 45% for acute injuries, compared with control group teams that performed their usual training. The preventive effect was more evident for male than for female players. There was no preventive effect on injury prevalence for overuse injuries.

**What are the findings?**

- The *Knee Control* programme reduced the incidence of acute injuries by 45% in youth floorball players.
- No preventive effect on prevalence of overuse injuries was seen.
- A possible adverse intervention effect was seen for overuse knee injuries in female players with two times higher prevalence rate in the intervention group.

**How might it impact on clinical practice in the future?**

- The *Knee Control* programme should be implemented as part of warm-up for floorball training in youth athletes.
- Teaching coaches how to instruct on correct exercise execution, how to provide feedback to players, and how to modify exercises if necessary, could help reduce potential adverse outcomes. This is important to ensure high adherence to *Knee Control*.
