# Supplementary material for: Forty-five per cent lower acute injury incidence but no effect on overuse injury prevalence in youth floorball players (aged 12–17 years) who used an injury prevention exercise programme: two-armed parallel-group cluster randomised controlled trial
Source: Br J Sports Med. 2020 Jan 28;54(17):1028–35. doi: 10.1136/bjsports-2019-101295 (PMC7456671; doi:10.1136/bjsports-2019-101295)
Supplement: Supplementary data [file bjsports-2019-101295supp001.pdf]

Supplementary table 1. Details of the running warm-up programme and *Knee Control* injury prevention exercise programme (IPEP) used in the intervention group

**Exercise**	**Instructions**	**Sets repetitions/ duration**
**Running exercises**	Course consists of 6-10 pairs of parallel cones, approximately 10 m wide and 20 m long. To be performed before each training session and match. Ensure good hip-knee-foot alignment during the running exercises	5 min
Running	Straight ahead	
Sideways jumps		
Running	With change of direction	
Sideways jumps	Jump with body contact; shoulder to shoulder	
Running	Alternate forwards and backwards running, with decelerations and accelerations	
***Knee Control* IPEP**		
**One legged knee squat**	Slow movement with smooth turn, stable hip with horizontal pelvic position and non-supporting foot in front of the body with slightly flexed hip and knee. Knee motion forwards in the sagittal plane, alignment with foot	
Level A	Hands on hips 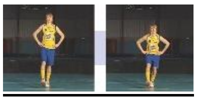	3×8-15 reps
Level B	Hold a stick 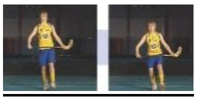	3×8-15 reps

Knäkontroll®, SISU Idrottsböcker©, Sweden, 2005

Level C	Dribble in front of you	3×8-15 reps
	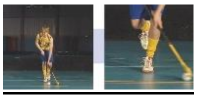	
Level D	Pass the ball against the court	3×8-15 reps
	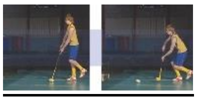	
Partner exercise	Both teammates stand on one leg with backs against each other. Hand over the floorball in different directions	3×8-15 reps
	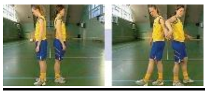	
**Pelvic lift**	Supine position; lift the pelvis from ground to neutral position while keeping the back straight	
Level A	Both feet on the ground and hands across the chest	3×8-15 reps
	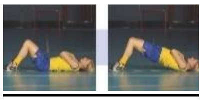	
Level B	Feet on a bench and hands across the chest	3×8-15 reps
	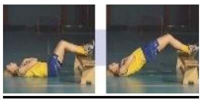	
Level C	One foot on the ground and the contralateral leg fixed straight, arms on the ground alongside the body. Lift the pelvis and abduct the straight leg	3×8-15 reps
	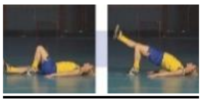	
Partner exercise	Supine position with feet towards each other. One foot on the ground and contralateral foot against the teammates foot	3×8-15 reps
	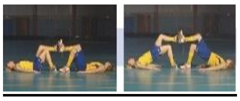	

Knäkontroll®, SISU Idrottsböcker©, Sweden, 2005

**Two legged knee squat**	Slow movement with smooth turn, back straight, and feet shoulder-width apart, pointing forwards and with soles in contact with the ground. Knee motion forwards in the sagittal plane, alignment with the foot. The trunk should be upright. Knee flexion to 90°.	
Level A	Hands on hips	3×8-15 reps
	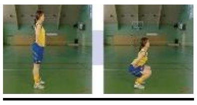	
Level B	Hold stick in front of the body with straight arms	3×8-15 reps
	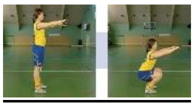	
Level C	Hold stick over the head with straight arms	3×8-15 reps
	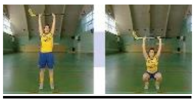	
Level D	Same as level C but continue movement and rise up on the toes after returning to the starting position and stay in the elevated position briefly	3×8-15 reps
	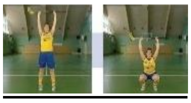	
Partner exercise	Teammate stands in front of you, facing towards you. Hold two sticks, one in each hand	3×8-15 reps
	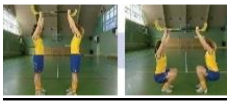	

Knäkontroll®, SISU Idrottsböcker©, Sweden, 2005

**The bench**	Lift body and keep it in a straight line, with no hip flexion/extension. Elbows placed beneath the shoulders with 90° flexion of the shoulders and elbows	
Level A	Prone position; support on knees and on lower arms with elbows kept beneath the shoulders 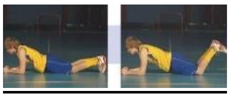	15-30 s
Level B	Same as level A but with support on the tip of the feet 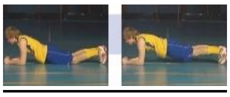	15-30 s
Level C	Same as level B, but raise one foot at a time up and down; alternate sides 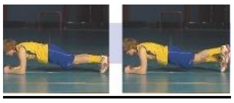	15-30 s
Level D	Lie sideways with support on the foot and lower arm with the elbow kept beneath the shoulder and the other hand on the hip; lift the hip off the ground and stay briefly in that position with good control before slowly returning to the starting position. Elbow under the shoulder with 90° shoulder abduction and 90° elbow flexion. No hip abduction/adduction 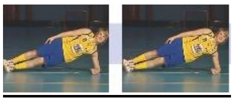	5-10 reps
Partner exercise	Same as level A but teammate face to face. Pass a floorball with the hand 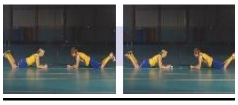	15-30 s

Knäkontroll®, SISU Idrottsböcker©, Sweden, 2005

**The lunge**	Take a deep step with a marked knee lift and soft landing; the rear knee should not touch the ground. Keep good knee-over-foot alignment. Trunk control maintained with minimum lateral movement. Stable hip with horizontal pelvic position. Forward knee flexed to 90°. Foot placed in the sagittal plane pointing forwards	
Level A	Hands on the hips; move forwards with each step 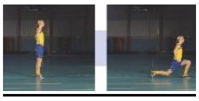	3×8-15 reps
Level B	Hold the stick in front of the body; move forwards with each step 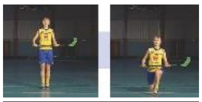	3×8-15 reps
Level C	Same as level B but continue the movement and rise up on the toes between each lunge 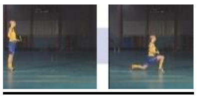	3×8-15 reps
Level D	Same as level B, rotate the upper body while stepping forwards and position the stick laterally to the front leg; move forwards with each step and alternate sides 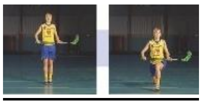	3×8-15 reps
Partner exercise	Teammate stands in front of you 5 m away; pass the floorball, perform a forward lunge, and receive a pass back 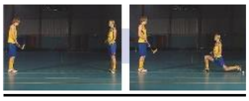	3×8-15 reps

Knäkontroll®, SISU Idrottsböcker©, Sweden, 2005

**Jump/landing**	Jump with a controlled and soft landing; stay briefly in the landing position. Keep good knee-over-foot alignment. Trunk control maintained with minimal motion. Knee control maintained with minimal wobble sideways. Foot placed in the sagittal plane pointing forwards at landing	
Level A	Stand on one leg with the knee slightly bent and hands on the hips; make a short forwards jump and land on the same foot; then jump backwards to the starting position	3×8-15 reps
	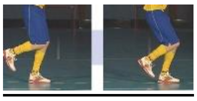	
Level B	Stand on both feet shoulder-width apart and hold a stick; make two forward jumps and land on one foot after the third jump with a controlled and soft landing; alternate sides	3×8-15 reps
	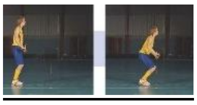	
Level C	Same as level B but rotate the body 90° before landing on one leg; alternate sides	3×5 reps
	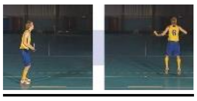	
Level D	Sideways jump on one foot and dribble with a ball and stick in front of you	3×5 reps
	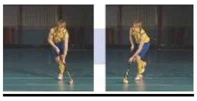	

Knäkontroll®, SISU Idrottsböcker©, Sweden, 2005

Partner exercise	Teammate stands in front of you approximately 5 m away; make sideways jump on one foot and pass the floorball to your teammate. Jump back to the starting position and receive a pass back 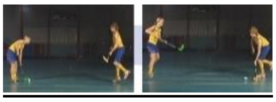	3×8-15 reps

Knäkontroll®, SISU Idrottsböcker©, Sweden, 2005
