# Supplementary figures and images for: Forty-five per cent lower acute injury incidence but no effect on overuse injury prevalence in youth floorball players (aged 12–17 years) who used an injury prevention exercise programme: two-armed parallel-group cluster randomised controlled trial
Source: Br J Sports Med. 2020 Jan 28;54(17):1028–35. doi: 10.1136/bjsports-2019-101295 (PMC7456671; doi:10.1136/bjsports-2019-101295)

Supplementary figure 1. Flowchart of the weekly survey.

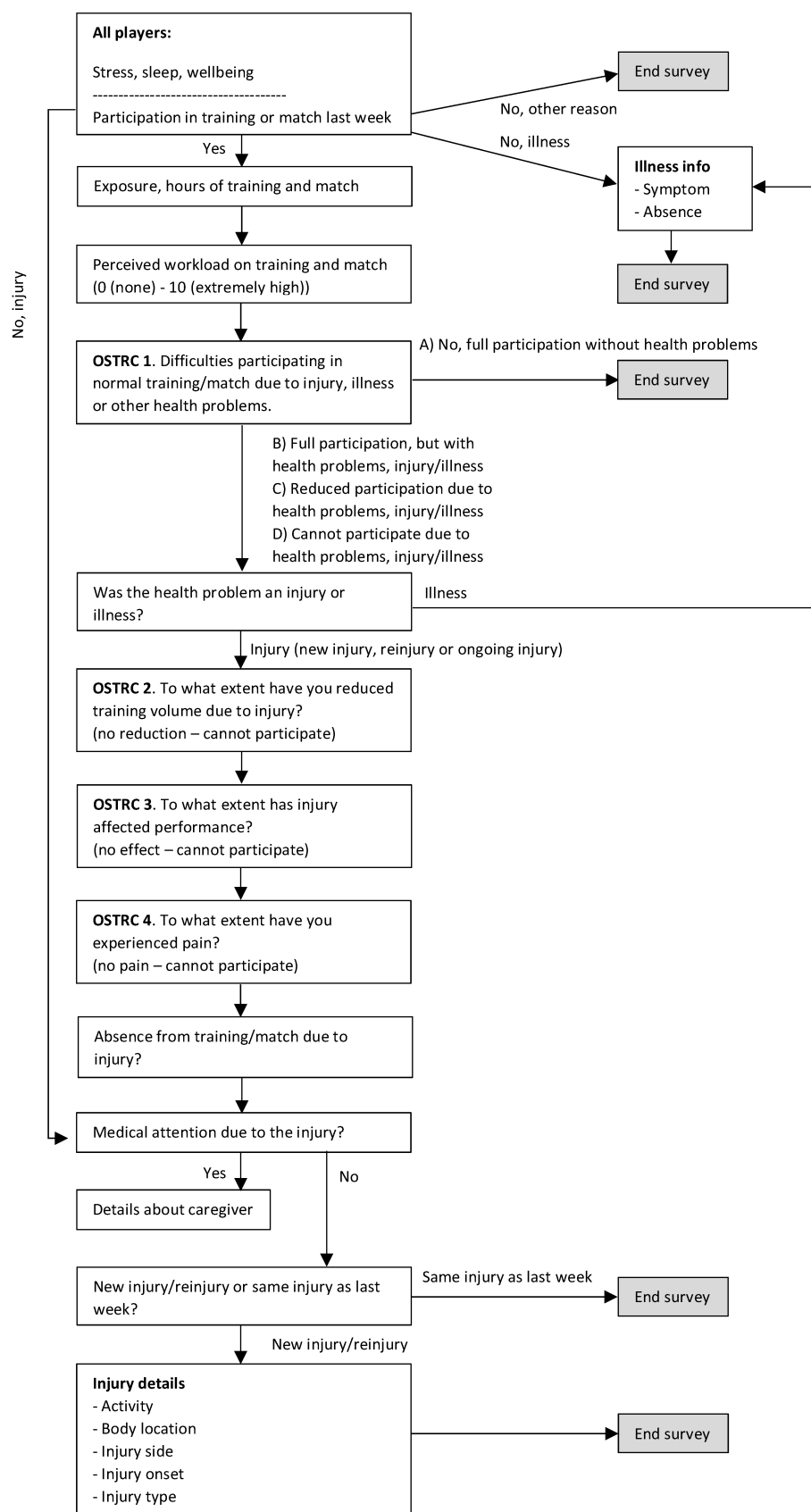

Supplement: Supplementary data [file bjsports-2019-101295supp002.pdf]
