# Supplementary material for: Forty-five per cent lower acute injury incidence but no effect on overuse injury prevalence in youth floorball players (aged 12–17 years) who used an injury prevention exercise programme: two-armed parallel-group cluster randomised controlled trial
Source: Br J Sports Med. 2020 Jan 28;54(17):1028–35. doi: 10.1136/bjsports-2019-101295 (PMC7456671; doi:10.1136/bjsports-2019-101295)
Supplement: Supplementary data [file bjsports-2019-101295supp003.pdf]

Supplementary table 2. Baseline characteristics for included players (n=471)

	**Intervention**			**Control**		
	**Total (n=301)**	**Female (n=107)**	**Male (n=194)**	**Total (n=170)**	**Female (n=35)**	**Male (n=135)**
Age (years), mean (SD)	13.6 (1.1)	13.8 (1.3)	13.5 (0.9)	13.2 (1.3)	13.6 (1.9)	13.1 (1.2)
Floorball experience (years), mean (SD)	5.1 (2.3)	4.4 (2.4)	5.5 (2.1)	4.6 (2.4)	5.1 (2.7)	4.4 (2.3)
Participating in other sports (yes), n (%)*	183 (61)	57 (53)	126 (65)	106(62)	23 (66)	83 (61)
Menarche (yes), n (%)†		75 (70)			18 (51)	
Sports profile in school (yes), n (%)	61 (20)	14 (13)	47 (24)	17 (10)	2 (6)	15 (11)
Previous experience of using *Knee Control* IPEP, n, (%)‡						
Regularly	44 (16)	19 (19)	25 (14)	17 (11)	2 (7)	15 (12)
Sporadically	81 (29)	28 (28)	53 (29)	41 (27)	13 (42)	28 (23)
No	158 (56)	52 (52)	106 (58)	96 (62)	16 (52)	80 (65)

*Currently participating in other sports missing for 13 players in the intervention group and 14 in the control group.

†Menarche missing for 3 girls in the intervention group and 2 in the control group.

‡Previous experience of using *Knee Control* IPEP missing for 18 players in the intervention group and 16 in the control group.
